# Autochthonous *Nocardia cerradoensis* Infection in Humans, Spain, 2011 and 2014

**DOI:** 10.3201/eid2201.150771

**Published:** 2016-01

**Authors:** Maria Ercibengoa, Emilio Pérez-Trallero, José Maria Marimón

**Affiliations:** University of the Basque Country Faculty of Medicine, San Sebastián, Spain (M. Ercibengoa, E. Pérez-Trallero);; Biomedical Research Center Network for Respiratory Diseases, San Sebastián (E. Pérez-Trallero, J.M. Marimón);; Hospital Universitario Donostia- Instituto de Investigación Sanitaria Biodonostia, San Sebastián (E. Pérez-Trallero, J.M. Marimón)

**Keywords:** Nocardia cerradoensis, respiratory infections, transmission, chronic obstructive pulmonary disease, COPD, chronic obstructive lung disease, Spain, autochthonous cases, human pathogen, immunocompromised persons, bacteria

## Abstract

*Nocardia cerradoensis* was first isolated in 2003 in the El Cerrado region of Brazil; since then, only 2 human infections, in France and Spain, have been reported. We describe 3 autochthonous cases in residents of Spain during 2011 and 2014. Together these cases support the idea of an emerging global pathogenic microorganism.

*Nocardia cerradoensis* was first described in 2003 from a soil sample collected from a cultivated field in the Brazilian Cerrado, a broad region of woodland savannah (*1*). However, the first human case of disseminated infection was not reported until 2015; the patient was an immunosuppressed woman in Rennes, France (*2*). Later in 2015, a case of human skin infection with *N. cerradoensis* was described in Spain (*3*).

Little is known about the transmission of *Nocardia* species, although it is assumed that the bacteria enter the body mainly by inhalation of contaminated dust. In fact, pulmonary nocardiosis, which mostly affects immunocompromised patients, is the most frequent clinical manifestation of *Nocardia* infection (*4*). We report 3 autochthonous cases of *N. cerradoensis* infection in humans in Spain and discuss the source of infection with this recently described human pathogen.

## The Study

The study was performed at Donostia University Hospital in San Sebastián-Donostia, the capital city of the province of Gipuzkoa in northern Spain, during 1992–2014. A total of 253 isolates of *Nocardia* species were obtained from 179 patients. Species identification was performed by sequencing 1,188-bp and 401-bp fragments of the 16S rRNA (*5*) and *hsp65* (65-kDa heat shock protein) (*6*) genes, respectively. A total of 3 *N. cerradoensis* isolates were identified; for these and other infrequently found species, we also analyzed 400-bp and 445-bp fragments of the *rpoB* (RNA polymerase B) (*7*) and *secA1* (essential secretory protein SecA1) (*8*) genes. We then identified similar gene sequences in GenBank by using blastn (http://www.ncbi.nlm.nih.gov/blast).

For the 3 *N. cerradoensis* strains, sequences of the 16S rRNA, *rpoB*, and *secA1* gene fragments had the best GenBank matches (>99% similarity) with corresponding genes of *N. cerradoensis* strains W9747 (accession no. NR117400) and DSM44546 (accession nos. JN215712 and JN042082). Sequences of the *hsp65* genes from the 3 isolates shared 99.9% similarity with each other but only 85.0% similarity with the *hsp65* gene sequence of *N. cerradoensis* strain DSM44546 (accession no. AY756519). Sequences from our study were submitted to GenBank (accession nos. KT749656– KT749667).

Antimicrobial susceptibility was determined by broth microdilution (Sensititer microtiter trays; Trek Diagnostics Systems, East Grinstead, UK), and results were interpreted according to Clinical and Laboratory Standards Institute guidelines (*9*). Pulsed-field gel electrophoresis (PFGE) was performed as described (*10*), except that restriction enzyme digestion was performed with *Xba*I.

The first *N. cerradoensis* strain isolated in Gipuzkoa was from a morbidly obese 67-year-old woman with stable, but severe, chronic obstructive pulmonary disease (COPD) that was being treated with corticosteroids and bronchodilators. On January 18, 2011, the woman had a routine follow-up visit with her general practitioner, during which a sputum sample was obtained. *Haemophilus influenzae* and *N. cerradoensis* were isolated from the sample, which was of good microbiologic quality (>25 leukocytes and no squamous epithelial cells per 10× magnification field). The patient’s COPD status was stable, so she was not given antimicrobial drugs for treatment of either of the 2 pathogens. However, on February 11, 2011, she was hospitalized in the study hospital’s pulmonary department because of an episode of respiratory failure in the context of an acute COPD exacerbation. No clinical specimens were obtained for microbiologic testing, but because *N. cerradoensis* was previously isolated from her sputum, the patient was given supportive care, inhaled tobramycin, and oral trimethoprim/sulfamethoxazole (160/800 mg) for 1 month, and the symptoms resolved. Sputum samples collected on February 14 and March 30 were negative for *Nocardia* species. Of note, the daughter of this patient had lived in El Cerrado for a number of years; however, the patient had not visited her daughter in Brazil before the isolation of *N. cerradoensis*, and the daughter had not returned to Spain in the year preceding isolation of *N. cerradoensis* from her mother.

The second *N. cerradoensis* strain was isolated on October 11, 2011, from a sputum sample of a 64-year-old man with a history of moderate COPD. He was a heavy smoker and had been using inhaled, short-acting bronchodilators and corticosteroids since 2001. The patient sought outpatient consultation at the study hospital because of cough and hemoptysis; he was afebrile. Tuberculin test results and cultures for mycobacteria were negative. The patient was empirically treated with amoxicillin/clavulanic acid. Sputum samples collected on October 29 and 30 were negative for *N. cerradoensis* and other potential pathogens.

The third *N. cerradoensis* strain was isolated from the sputum sample of an 82-year-old man with pulmonary emphysema. On October 16, 2014, the man sought care in the study hospital’s emergency department for increasing dyspnea of a few days’ duration that had not improved despite treatment with corticosteroids and levofloxacin (500 mg 1×/d for 3 days). The patient had frequent coughing with yellowish expectoration but no fever; he received a diagnosis of respiratory infection and alteration (worsening) of his COPD status. Thoracic scan results showed cylindrical bronchiectasis and subpleural consolidations in both lungs; culture of a sputum sample obtained on October 22 was positive for *N. cerradoensis* and *Aspergillus fumigatus*. Because of the patient’s clinical status, the *A. fumigatus* was considered a colonizer (i.e., present without causing active disease). He was hospitalized and given trimethoprim/sulfamethoxazole (160/800 mg 2×/d for 30 days) for treatment of the *N. cerradoensis* infection. Cultures of sputum samples collected on December 3 and 16 were negative for *Nocardia* species.

The 3 *N. cerradoensis* isolates showed the same antimicrobial susceptibility pattern: nonsusceptibility to amoxicillin/clavulanic acid, minocycline, and ciprofloxacin (MICs >64/32 μg/mL, >4 μg/mL, and >8 μg/mL respectively) and susceptibility to ceftriaxone, imipenem, linezolid, amikacin, and trimethoprim/sulfamethoxazole (MICs <8 μg/mL, <0.25 μg/mL, 1 μg/mL, <0.25 μg/mL, and <0.5/9.5 μg/mL, respectively). After *Xba*I digestion, isolates from the first 2 patients had indistinguishable PFGE patterns that differed from that of the third patient’s isolate (Figure). However, no epidemiologic relationship could be established between the first and second patients: they lived in different villages (≈50 km apart) and had not been hospitalized nor been in the hospital’s outpatient clinic or emergency department at the same time.

**Figure:**
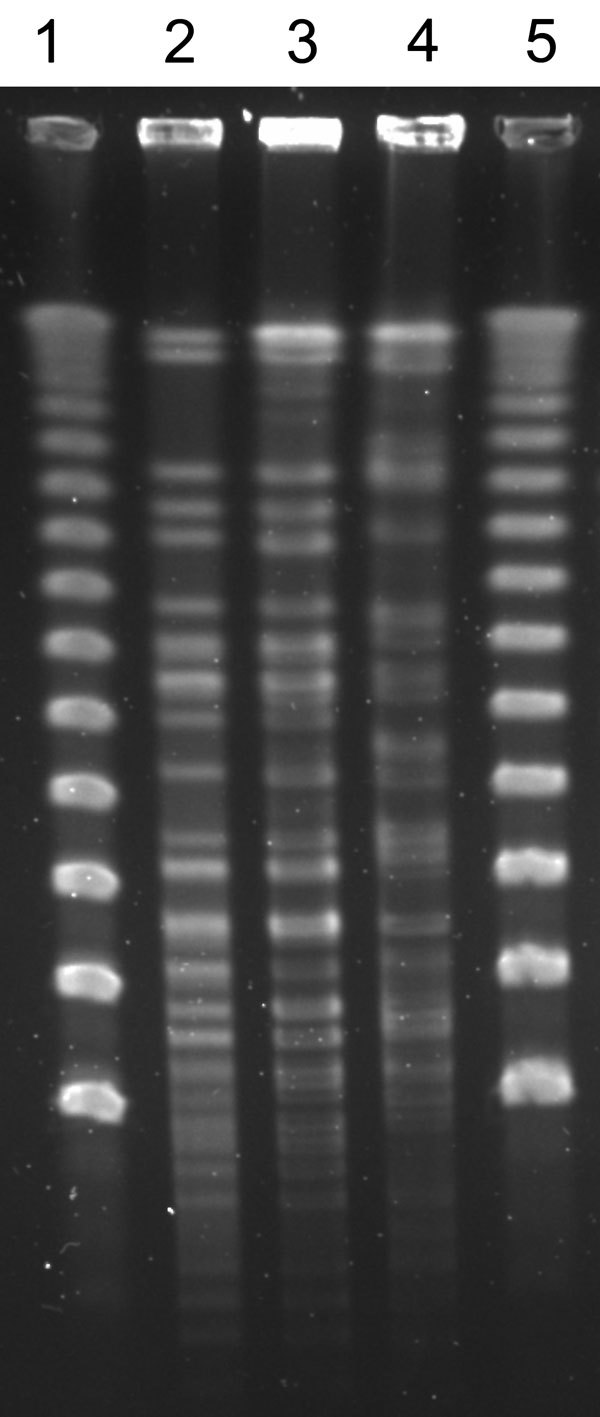
Pulsed-field gel electrophoresis patterns of *Nocardia cerradoensis* isolates (after *Xba*I restriction enzyme digestion) from 3 chronic obstructive pulmonary disease patients in Gipuzkoa, northern Spain. Lanes 1 and 5, DNA molecular weight marker (50-kbp ladder). Lanes 2, 3, and 4, isolates corresponding to patients 1, 2, and 3, respectively.

Because of the environmental origin of *Nocardia* species, their isolation, particularly in respiratory samples, might indicate colonization rather than clinical infection. In these cases, *N. cerradoensis* was isolated from the bronchial secretions of 3 patients who were at high risk for development of *Nocardia* clinical infection because they were immunosuppressed as a consequence of long-term corticosteroid therapy for COPD. The first and third patients received a diagnosis of pulmonary nocardiosis and were specifically treated with trimethoprim/sulfamethoxazole, with symptom resolution. However, the physicians in charge considered the presence of *N. cerradoensis* in the second patient to be a colonization rather than active disease. This diagnosis was reinforced by the quick disappearance of the pathogen from respiratory samples without specific treatment (the patient received only a short course of amoxicillin/clavulanic acid, to which *N. cerradoensis* was resistant).

## Conclusions

*N. cerradoensis* has not been isolated from environmental samples from any part of Europe. The first *N. cerradoensis* isolate detected in Gipuzkoa was from a patient whose daughter lived in the El Cerrado region of Brazil. However, 2 facts point to local acquisition of the pathogen: the patient and her daughter had not had personal contact during the year preceding the isolation of *N. cerradoensis*, and PFGE patterns were identical for the isolates from this patient and the second patient, with whom she had no contact. Our finding of human infections with *N. cerradoensis*, together with the reported cases from France and from a different city in Spain (*2**,**3*) support the idea of an emerging global pathogenic microorganism.
